# Patient Explanation of Adherence and Non-Adherence to Venous Leg Ulcer Treatment: A Qualitative Study

**DOI:** 10.3389/fphar.2021.663570

**Published:** 2021-06-03

**Authors:** Carolina D. Weller, Catelyn Richards, Louise Turnour, Victoria Team

**Affiliations:** Monash Nursing and Midwifery, Faculty of Medicine, Nursing and Health Sciences, Monash University, Melbourne, VI, Australia

**Keywords:** venous leg ulcer, patient experience, evidence-based guidelines, Theoretical Domains Framework, adherence—compliance—persistance

## Abstract

The aim of this study was to understand which factors influence patients’ adherence to venous leg ulcer treatment recommendations in primary care. We adopted a qualitative study design, conducting phone interviews with 31 people with venous leg ulcers in Melbourne, Australia. We conducted 31 semi-structured phone interviews between October and December 2019 with patients with clinically diagnosed venous leg ulcers. Participants recruited to the Aspirin in Venous Leg Ulcer Randomized Control Trial and Cohort study were invited to participate in a qualitative study, which was nested under this trial. We applied the Theoretical Domains Framework to guide the data analysis. The following factors influenced patients’ adherence to venous leg ulcer treatment: understanding the management plan and rationale behind treatment (Knowledge Domain); compression-related body image issues (Social Influences); understanding consequences of not wearing compression (Beliefs about Consequences); feeling overwhelmed because it’s not getting better (Emotions); hot weather and discomfort when wearing compression (Environmental Context and Resources); cost of compression (Environmental Context and Resources); ability to wear compression (Beliefs about Capabilities); patience and persistence (Behavioral Regulation); and remembering self-care instructions (Memory, Attention and Decision Making). The Theoretical Domains Framework was useful for identifying factors that influence patients’ adherence to treatment recommendations for venous leg ulcers management. These factors may inform development of novel interventions to optimize shared decision making and self-care to improve healing outcomes. The findings from this article will be relevant to clinicians involved in management of patients with venous leg ulcers, as their support is crucial to patients’ treatment adherence. Consultation with patients about VLU treatment adherence is an opportunity for clinical practice to be targeted and collaborative. This process may inform guideline development.

## Introduction

Venous leg ulcers (VLU) are chronic skin ulcers and are the most common chronic wound of the lower limbs in Australian primary care settings (Pacella et al., 2018). VLU are classified as C6 stage of a chronic venous disorder, as per the updated Clinical (C) version of Clinical-Etiology-Anatomy-Pathophysiology (CEAP) classification (Lurie et al., 2020). They are caused by chronic venous insufficiency due to persistent high blood pressure in varicose veins (Meulendijks et al., 2019; Weller et al., 2019). Best practice management recommendations for people with VLU are compression, leg elevation, physical exercise and adequate nutrition (Australian Wound Management Association New Zealand Wound Care Society, 2011).

The estimated VLU prevalence globally is between 1 and 3% of people (Graves and Zheng, 2014b; Domingues et al., 2018; Xie et al., 2018). Chronic wounds represent a considerable social and economic burden (KPMG, 2013; Graves and Zheng, 2014a), with an estimated annual cost of AUD $3 billion in Australia (Graves and Zheng, 2014), $32 billion in the United States (Nussbaum et al., 2018) and £50 million in the United Kingdom (Guest et al., 2017). Despite this burden, there is no international consensus on the best strategies for prevention and management of VLU (Franks et al., 2016; Weller et al., 2019). To address this health issue in Australia, the Australian and New Zealand Clinical Practice Guidelines (CPG) for the prevention and Management of Venous Leg Ulcers (Australian Wound Management Association New Zealand Wound Care Society, 2011), (herein referred to as VLU CPG) was published in 2011. Despite the development and dissemination of VLU CPG (Australian Wound Management Association New Zealand Wound Care Society, 2011), a significant number of VLU remain unhealed (Franks et al., 2016), warranting further consideration of contributing factors to develop solutions to this silent epidemic (Pacella et al., 2018). The challenges faced when managing people with VLU are not unique to Australia (Franks et al., 2016). Compression use, as an example, has been reported as being problematic across Switzerland (Probst et al., 2020) and the United Kingdom (Guest et al., 2018).

Current best practice recommendations include assessment and diagnostic evaluation of the lower limb to exclude arterial causes prior to compression application (Weller et al., 2018), including venous ultrasound and/or assessment of Ankle Brachial Pressure Index (ABPI). Once arterial insufficiency is excluded, below knee multi-layer compression therapy is recommended (Andriessen et al., 2017; O'Meara et al., 2013). In instances where there is unclear aetiology or if the ulcer is not healed after three months of treatment, VLU CPG recommends the patient be referred to a specialized wound clinic (Australian Wound Management Association New Zealand Wound Care Society, 2011). In addition to recommendations surrounding assessment and compression, VLU CPG (Australian Wound Management Association New Zealand Wound Care Society, 2011) distinguishes the importance of preparing the VLU patients sufficiently and enabling them to co-participate and share in decision making in wound management (Ritchie and Taylor, 2018). Patients with VLUs would usually consult their GP in the first place. The support-seeking pathway that VLU patients are supposed to follow includes initial consultation by a GP, who should perform clinical assessment, including vascular assessment to exclude arterial involvement. In absence of arterial involvement, compression therapy should be prescribed and other recommendations provided, including leg elevation, adequate nutrition, pain management and physical exercise, as indicated in the guidelines (Australian Wound Management Association New Zealand Wound Care Society, 2011). In absence of healing improvement over three months, patients should be referred to tertiary clinic for assessment. Clinicians’ limited awareness about these guidelines, reliance on their clinical experience and suggestions from colleagues rather than clinical guidelines, and their limited skills in ABPI measurement and health system organisational issues, including absence of duplex ultrasound in some practices, lead to suboptimal management of patients with VLUs (Weller et al., 2019; Weller et al., 2020).

Optimal VLU treatment is collaborative, with patients tasked with specific self-care actions at home after VLU care tasks are demonstrated by clinicians in the clinical setting. These tasks may include, although will not be limited to compression application. Although patients usually attend regular scheduled review appointments, wound care is often done at home independently (Walburn et al., 2017). Current management of VLU continues to challenge patients and clinicians with reports of suboptimal VLU treatment (Franks et al., 2016). A recent study of data from the Bettering the Evaluation and Care of Health (BEACH) program reported only 2.1% of VLU patients are treated with compression (Gethin et al., 2020). Furthermore, another study reported greater proportion of health professionals were unaware of the VLU CPG (Weller et al., 2020). Australia has limited available means for monitoring patterns and quality of care for people diagnosed with VLU as there is no clinical VLU registry (Weller and Evans, 2014) and, as a result, there is variation in the way VLU is managed in primary care settings. Clinician barriers to using and implementing evidence-based interventions for VLU treatment include limited awareness of the VLU CPG, organisational barriers, and suboptimal knowledge and skills of VLU management (Weller et al., 2019; Weller et al., 2020).

Clinicians reported patient adherence to VLU management plans was a key area of concern (Weller et al., 2020), specifically the application and long term use of compression therapy (Probst et al., 2019; Probst et al., 2020). Compression discomfort or pain (Boxall et al., 2019) can impact on compression adherence. Insufficient understanding of the benefits of compression treatment may contribute to non-adherence (Jull et al., 2004; Probst et al., 2020). The consequences of non-adherence to treatment recommendations include chronicity of VLUs, increased recurrence rate (Finlayson et al., 2018), infection (Bui et al., 2019) and secondary squamous cell carcinoma (Reich-Schupke et al., 2015). Disease progression (Meulendijks et al., 2020) and associated complications exacerbate wound pain (Leren et al., 2020), increase wound exudate, and reduce patients’ mobility, which in turn contributes to disease progression (Meulendijks et al., 2019), leads to premature disability (Mallick et al., 2020), and inadvertently influences patients’ quality of life (Barnsbee et al., 2019). Management of these complications require complex interventions and highlights the need for optimal care for this underestimated condition.

Adherence to evidence-based VLU management is not limited to compression use; education based interventions may improve adherence to leg elevation (Brooks et al., 2004) and therapeutic physical exercises (Kelechi et al., 2014). Despite this, clinician advice and education on VLU management is offered to less than 3.6% of VLU patients who attended primary health settings (Gethin et al., 2020). Few studies have examined the patient experience of adhering to VLU treatment (Weller et al., 2016) and patient education needs have yet to be fully understood (Shanley et al., 2020). Understanding patients’ behavior related to treatment adherence is important in planning and developing effective evidence-based interventions on VLU management (Presseau et al., 2019); and high-quality qualitative studies are required in order to understand patient behavior and to develop successful interventions. The aim of this study was to understand which barriers and enablers influence patients’ adherence to venous leg ulcer treatment recommendations in primary care settings.

## Materials and Methods

### Aim/s

The aim of this study was to understand barriers and enablers influencing patients’ adherence to venous leg ulcer treatment recommendations in primary care settings.

### Design

We adopted a qualitative study design, and conducted 31 semi-structured phone interviews with VLU patients, who had participated in the Aspirin in Venous Leg Ulcer (ASPiVLU) clinical trial (Weller et al., 2016). The ASPiVLU study was a randomized, double-blinded, multicentre, placebo-controlled trial, which investigated if daily oral dose of aspirin (300 mg), as an adjunct to compression, improved time to healing for clinically diagnosed VLU patients (Weller et al., 2016). The study participants were aged 18 years and older with one or more VLUs in the presence of venous insufficiency confirmed by clinical assessment and/or duplex ultrasound. Other eligibility criteria included: 1) the target ulcer, was separated from other VLUs by at least 1 cm; 2) was present for at least six weeks 3) target ulcer area measured by digital planimetry techniques was between ≥1 cm^2^ to ≤20 cm^2^; 4) Ankle brachial pressure index (ABPI) was  ≥ 0.7 mmHg or systolic toe pressure ≥ 50 mmHg to exclude arterial insufficiency; 5) the patient was able to provide informed consent (as per the medical practitioner’s clinical judgement). The participants were randomized to receive either aspirin or placebo for 24 weeks, adjuvant to compression. Time to healing within 12 weeks was the primary outcome of this study. Secondary outcomes were ulcer recurrence, wound pain, quality of life and wellbeing, adherence to compression therapy, adherence to study medication, serum inflammatory markers, number of hospitalisations, and adverse events at 24 weeks.

### Sample/Participants

Seventy-two participants from the ASPiVLU study, who expressed their interest to participate in other studies, were given a phone call by the trial coordinator and invited to participate in this nested qualitative study. Nine people said that they were no longer interested in research participation without explaining the reason. Seven people declined because they were too busy working and traveling. Nine people said that they would be unable to participate in the phone interview due to hearing problems. Eight people were too sick. Their partners/carers said that their health deteriorated and provided the following main health issues: mental health issues, dementia, and a recent fall. Thirty-nine participants confirmed their interest to participate and agreed to be interviewed. Interviews with all participants that agreed to be interviewed were scheduled at a convenient for them time.

Data saturation was reached on 25th interview, while some voice files were still with transcription services. We considered we reached saturation when subsequent analysis did not generate any new TDF construct. In total, we interviewed 31 participants. Participants were reimbursed with a $25 Coles Myer gift-card for participation. The trial coordinator contacted remaining people who agreed to be interviewed to thank them for their interest in study participation and inform that we were no longer recruiting participants.

### Theoretical Framework

To understand patients’ behavior related to VLU management adherence we were guided by the Theoretical Domains Framework (TDF) (Michie et al., 2005). This framework provides a theory-informed method for analyzing determinants of behavior. The TDF comprises a collection of domains, the product of a consensus strategy to be used in implementation research (Michie et al., 2005). The first version of this framework was developed in 2005 (Atkins et al., 2017), and later appraised and redeveloped in 2017 (Haardörfer, 2019). The 2017 version (Atkins et al., 2017) of the TDF framework includes 14 domains which guided our research. The TDF domains cover Knowledge, Skills, Beliefs about Capabilities, Beliefs about Consequences, Environmental Context and Resources, Social Influences, Behavioral Regulation, Optimism, Emotions, Goals, Social/Professional Role and Identity, Reinforcement, Intentions and Memory, Attention, and Decision Making.

### Data Collection

Data were collected, using the interview guide developed by CW and VT, and refined by LT and CW. The interview guide consisted of open-ended questions loosely matching the TDF domains (2017 version), and did not cover all 14 TDF domains (Table 1). These questions were followed by a series of prompts that probed barriers and enablers influencing patient adherence or non-adherence to VLU treatment recommendations (Michie et al., 2005). Interview questions were able to be modified by the interviewer depending on the flow of the interview. The interview guide was not piloted.

**TABLE 1:** The interview guide: Selected questions and matched theoretical domains.

Question	Prompt	Theoretical domain
If a participant used compression		
What factors or situations helped you in making a decision to adhere to compression therapy?	How did you find out about this as a treatment option?	Knowledge
What was the rationale/pros and cons for having compression therapy explained to you?	What were the benefits and negative aspects of using compression?	Beliefs about consequences
	How did you come to this decision?	Memory, attention, and decision processes
What feelings or emotions did you feel when deciding to use and to adhere to compression?		Emotion
How confident do you feel to self-manage compression application at home?		Skills
Describe your support network during this experience		Social influences
Previous experience: Had you adhered to compression therapy in the past if you have history of VLU’s?	What type of compression is the most comfortable? Why do you prefer this one?	Memory, attention, and decision processes
If a participant did not use compression		
How compression impacts on healing outcomes? Did anyone explain to you the importance of wearing compression?	Who? Health professionals, family, friends, ASPiVLU researchers?	Knowledge, social influence
	What was discussed?	
What factors helped you in making a decision to not use compression?	Explain how you came to the decision.	Memory, attention and decision processes
	What were the main influences on your decision?	

Data collection took place between October 2019 and December 2019. Phone interviews were conducted by two experienced researchers (LT and CR) from the University office. All interviews were audio recorded, using a handheld recording device. Participant verbal consent was obtained prior to recording. The average interviewing time was 35 min, ranging between 12 and 87 min, depending on the participants availability and their willingness to discuss the details of VLU management. The audio files were transcribed verbatim using a professional transcription service. Transcripts were checked alongside voice files by LT and CR to ensure the accuracy of the transcribed text. The interview transcripts were not sent back for the participants’ verification.

Participant and VLU characteristics data were collected as part of the ASPiVLU RCT study. A comprehensive overview of participant details and wound characteristics enabled a comparison of the VLU participant group of this study and VLU patients in the wider community.

### Ethical Considerations

This study was conducted in line with the ethical guidelines of the 1975 Declaration of Helsinki. The University Human Research Ethics Committee approval was obtained for both the main study and a nested qualitative study. Site-specific approvals from the participating tertiary specialist clinics were received from the participating health services ethics committees.

### Data Analysis

The data were analyzed using NVivo Version 12. We adopted a theory-driven conceptual analysis (Haardörfer, 2019) and utilized the TDF domains and constructs to guide our analysis (Atkins et al., 2017). Our coding framework was based on the TDF. CR conducted initial coding of the first three transcripts and developed the coding framework. The coding framework was then reviewed by VT and CW. Technically, utterances were mapped across a coding framework based on the TDF domains and associated constructs. We focused on barriers and enablers that influenced VLU patients’ decision to adhere to treatment recommendations in their care plan, which were then allocated to the relevant domains.

## Results

### Participant Characteristics

Thirty-one participants were included in this study: Sixteen participants (52%) were male and 15 (48%) were female. The mean age of participants was 64 years, ranging from 39 to 89. Medical history/comorbidities included hypertension (*N* = 19; 61%), osteoarthritis (*N* = 14; 45%), knee/hip surgery (*N* = 11, 35%), venous surgery/ligation (*N* = 10; 32%), type 2 diabetes (*N* = 10; 32%), rheumatoid arthritis (*N* = 5; 16%), deep vein thrombosis (*N* = 4; 13%), depression (*N* = 4; 13%), anaemia (*N* = 3, 10%). Additional socio-demographic details of the participants can be accessed in Table 2.

**TABLE 2:** Participant characteristics (*n* = 31).

Gender (number, percentage)		
Male	16	52%
Female	15	48%
Age		
<45	2	6%
45–54	2	6%
55–64	6	19%
65–74	12	39%
75–84	5	16%
85+	4	13%
Education		
Up to high school graduation	19	61%
Community college or Bachelor’s degree	8	26%
Postgraduate or professional degree	4	13%
Prefer not to answer	0	
Employment status		
Retired	24	77%
Working full-time (>30 h per week)	3	10%
Working part-time (<30 h per week)	1	3%
Self-employed	0	
Long term disability	1	3%
No response	1	3%
Not working	1	3%
Self-rated health		
Excellent/very good	4	13%
Good	19	61%
Poor	7	23%
Worse than poor	0	
No response	1	3%
Medical history		
Hypertension	19	61%
Osteoarthritis	14	45%
Knee/Hip surgery	11	36%
Venous surgery/Ligation	10	32%
Type 2 diabetes	10	32%
Rheumatoid arthritis	5	16%
Deep vein thrombosis	4	13%
Depression	4	13%
Anaemia	3	10%

### Summary of the Theoretical Domains Framework Domains

**FIGURE 1:**
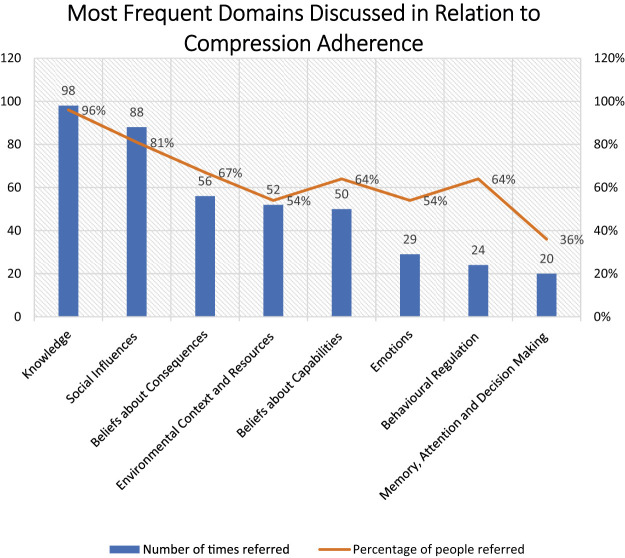
Most frequent domains discussed in relation to compression adherence.

**FIGURE 2:**
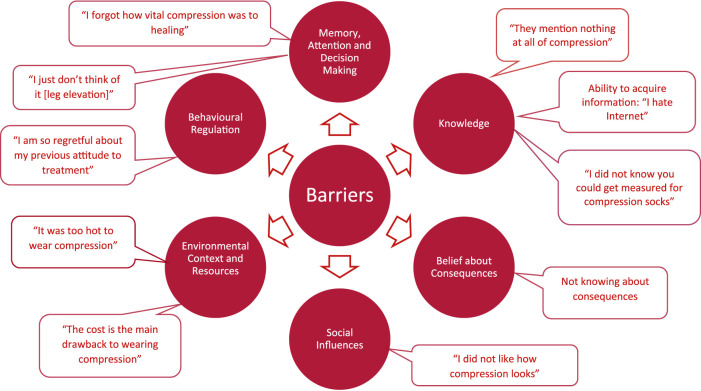
Barriers to patients’ adherence to the venous leg ulcer treatment recommendations.

**FIGURE 3:**
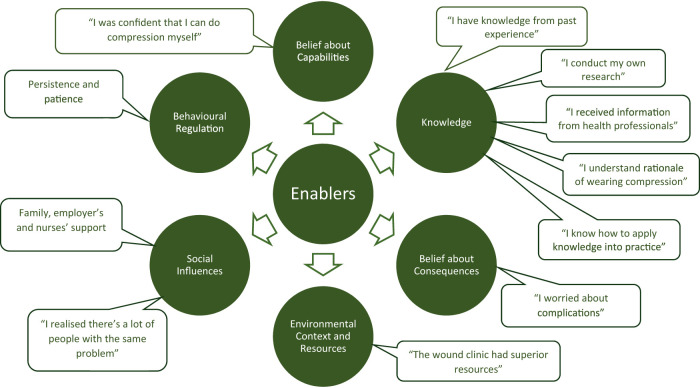
Enablers to patients’ adherence to the venous leg ulcer treatment recommendations.

Patients described the key aspects of VLU treatment process, including compression, appointment attendance, health information, leg elevation and nutrition. Of these, compression was the most frequently discussed recommendation: 64 times by 30 participants (Figure 1). The discussed barriers and enablers (Figures 2, 3) to treatment adherence discussed by participants related to the following domains: Knowledge (referred 98 times by 96% of participants), Social Influences (referred 88 times by 81% of participants), Beliefs about Consequences (referred 56 times by 67%), Emotions (referred 29 times by 54%), Environmental Context and Resources (referred 52 times by 54%) and Beliefs about Capabilities (referred 50 times by 64%), Behavioral Regulation (referred 24 times by 64%) and Memory, Attention and Decision Making (referred 20 times by 36%). Reinforcement, Intentions and Optimism, Skills, Goals, and Social/professional Role and Identity domains were not discussed by participants since they were not prompted to do so (Tables 3, 4).

**TABLE 3:** Barriers to patients’ adherence to the evidence-based guideline recommendations for venous leg ulcer management.

Domains	No of participants mentioned	No of times referred	Main constructs	Main themes	Sample quotes
Knowledge	23	32	Knowledge	Inadequate information given	*Q: Yeah. And they didn’t suggest to you about wearing compression then when you were going for dressing?*
					*A: Well, not at all. Nothing. They didn’t mention nothing at all of compression.* [P11]
					*They did suggest compression, but they didn't enforce it as absolutely necessary then. And they used different ways of cleaning the wound and dressing the wound. That was just interesting to see. They weren't really that experienced in dealing with those thing* [P19]
					*Q: So, they gave you a lot of information on the benefits that you said before. Had you ever heard about, besides the Tubigrip, had anyone else ever talked to you about compression, or the use of it in your management of your ulcers, or the care?*
					*A: They talk to you about compression, but I didn’t really know you could go and get measured for the socks for compression, which I’ve got. But the Comprilan is even better than the hand-made compression stockings* [P01]
					*Q: So, did you always feel like you had enough information about compression? Or…*
					*A: Yeah. Since I've started going to (the specialist wound clinic), yes.*
					*Q: Yeah.*
					*A: From original years ago, when I went to the hyperbaric clinic? No.*
					*Q: Okay. And what makes you say not so much with the earlier venous leg ulcer?*
					*A: No, they didn't—they just sort of fixed it and sent me on my way. They didn't give me a maintenance plan or anything really. They just said, “Just keep wearing this Tubigrip every day. Take it off at night, and,”—but they never said, “Make sure it's a new one every day,” or—yeah, they never gave me a lot of information at all really, no* [P31]
				Ability to acquire information	*Q: So, do you use the Internet at all to look for information?*
					*A: No, I hate it* [P04]
Social influences	19	44	Modelling		*Q: Did they say that you might have to keep wearing them (compression stockings) in the future or anything like that? Have they got a plan?*
					*A: That was funny though because (the GP) was sort of like, yeah, you can stop wearing them you don't have to worry about them anyway. But the doctors and the GPs in the wound clinic were all saying that I was going to have to wear it for the rest of my life. So is a bit of a mixed message there* [P19]
			Social norms	I didn’t like how the compression looks	*Q: Okay. And when was that, that you were wearing the stockings?*
					*A: Ah, I didn’t wear it for very long. And I can’t remember how long.*
					*Q: Okay. Was that—when you say you didn't wear it for very long, was that because it was uncomfortable? Because the doctor asked you to stop wearing them? Or A: No, I decided not to, just for vanity. I couldn’t stand the look of them* [P04]
					*A: I still wear them (compression stockings) now and then but I should wear them all the time... but I feel like an old man. There’s something about them, I don’t know. They're daggy* [P11]
					*A: Oh, summertime, I like to wear shorts and like the beach and stuff like that, and then I don't want to be wearing bandages on my legs, so I guess that's part of the reason why I stopped doing it as well. Getting looks fine when I wear jeans and stuff more. I don't mind so much, but it's just seems like a pain. No, just gets in the way, which is frustrating* [P19]
					*I’m maybe a bit self-conscious wearing it then. But I wear it all, all the other times, when I’m wearing long trousers* [P23]
					*Q: Did you have any emotions or were you concerned about doing compression when they said you had to do it?*
					*A: Ah it's—what'd you call it? Out of vanity, I don’t like wearing—I don't like having one thing on my legs. I look like a - yeah, it's self - a self-conscious thing, I suppose you call it*? [P31]
Beliefs about consequences	20	35	Consequence	Worried about health complications	*Q: I’m still looking what factors might have pushed you to say, “I have to wear this (Compression)”*
					*A: … Well; I just saw the consequences of not wearing it.*
					*Q: … what are the consequences if you don't wear all it?*
					*A: Well, to be less healing than … I could see my veins were in a terrible state and that they were swollen, and I could see that it would help keep them … The compression would help with the swelling right down from my side to the ankle, and especially around the knee area, above and below the knee, there were very pronounced and worsening veins, so I just thought that would help. Also, I didn't like looking at everything in that state. The stocking could have helped keep it all tight. I'm not talking about how it looked, because I felt it might help the veins from popping out [P17]*
					*My sister is recently retired, a carer for (facility name removed for privacy), and it was at Christmas time I just said to her, “Look at my leg. It’s getting worse. Look at it. It’s awful”, and she said to me, “Look, you really should be trying to put a compression stocking or something on it”, and she was trying to get me to put something on it to keep the fluid out of my leg, and that’s the best information I possibly had from her because she’d seen them on elderly people, and I was saying to her, “But I’m not old. I shouldn’t have this happening to my leg”, and then I started to get really concerned, and I thought, “I have to look for a solution because it’s not getting any better, it’s getting worse”, and you become quite desperate because your legs have to hold you, and I was terrified someone was going to hit me or bump me [P28]*
Emotions	16	26	Stress	I felt self-conscious	*Q: Okay. And when was that, that you were wearing the stockings?*
					*A: Ah, I didn’t wear it for very long. And I can’t remember how long.*
					*Q: Okay. Was that—when you say you didn't wear it for very long, was that because it was uncomfortable? Because the doctor asked you to stop wearing them? Or -A: No, I decided not to, just for vanity. I couldn’t stand the look of them.* [P04]
					*Q: No, tell me about it. I know. And so you said before that about compression, so who sort of instructed or gave you information about compression to start?*
					*A: One of the doctors at (Location). I still wear them now and then but I should wear them all the time... but I feel like an old man. There’s something about them, I don’t know. they're daggy* [P11]
					*Q: And how did you feel about compression to begin with? I know you mentioned that you sort of do, you stopped doing it for a little bit. What was it that made you decide?*
					*A: Oh, summertime, I like to wear shorts and like the beach and stuff like that, and then I don't want to be wearing bandages on my legs, so I guess that's part of the reason why I stopped doing it as well. Getting looks fine when I wear jeans and stuff more. I don't mind so much, but it's just seems like a pain. No, just gets in the way, which is frustrating* [P11]
					*Q: How did you feel when deciding to use or like—adhere to the compression treatment?*
					*A: Well, it doesn’t worry me because I’ve used it … [inaudible], being a bit self-conscious but I’ve usually got jeans on anyway, but when I’m wearing shorts around that or thongs I’ll make sure I don’t wear the compression bandage, stockings—yeah.*
					*Q: You won’t wear it?*
					*A: I don’t wear it. Not when I’ve got thongs on.*
					*Q: Okay. Why is that? Is it because they’ve got close toes or?*
					*A: No, they haven’t … I’d say open toes but no I just don’t I’m maybe a bit self-conscious wearing it then. But I wear it all, all the other times, when I’m wearing long trousers* [P23]
				Frustration	*At the time, when there was this total irregularity and an inconsistency of treatment … I was very irritated indeed* [P21]
					*Q: And you felt secure with that? Did you ever doubt them?*
					*A: Not really, I suppose the only thing is I might have got a bit frustrated when it didn’t heal as quickly as I wanted it to* [P26]
Environmental context and resources	19	43	Environmental stressors	Too hot to wear compression	*Q: And how did you feel about doing compression therapy? The bandage?*
					*A: The bandages are awful especially when it's 40 degrees outside, it's the last thing you feel like wearing but you have to do it. Sorry, you just have to do it* [P14]
					*If my feet are fat or swollen I should say, and I’m on my feet a lot. It was a bit hard in summer because I got too hot, I’ve got wool pants on and these stockings, it was just too hot. But yeah just so I wouldn’t have swollen feet, it’s a seven-hour shift and I’m on my feet for six hours, so I wear them then* [P25]
					*Q: Okay. And do you wear the compression stockings now?*
					*A: No, I don't.*
					*Q: Okay.*
					*A: I should, but I don't.*
					*Q: When you say you should, was that something that the A: Well, oh you know they said, “You should wear the compression stockings,” and I do - in the winter time, I do wear them.*
					*Q: Yes.*
					*A: But in the summertime, I just want - at first, it was too hard to put on. And they're too hot* [P30]
			Resources/material resources	Cost	*Q: Yes, probably you would be able to use the other one with the Velcro as opposed to the pull-up ones (compression hosiery)?*
					*A: Yeah. But there’s issues with … no, they’re very expensive, although I was told about it.* [P13]
					*Q: Is there any other drawbacks to wearing compression vs. the Tubi vs. …*
					*A: The cost* [P28]
Beliefs about capabilities	20	35	Perceived competence	Other health issues	*Q: Yeah. Or why do you think it wasn't quite healing? Did the doctors say anything about that?*
					*A: Uh, I have other health issues that probably contributed. I think they did take that into account, and my immune system is deficient.*
					*Q: And so did that sort of, do you think prevented healing or it prevented you from doing these exercise?*
					*A: yeah, had a lot to do with it. Yeah, definitely* [P14]
Behavioral regulation	14	24	Breaking habit	Commitment to treatment	*Q: Did you have anything else that you wanted to add in? Anything that we might have missed about venous leg ulcer treatment?*
					*A: Not specifically about the treatment. It was my attitude to the treatment that I'm so much regretful about. I should have shown more appreciation of it, as it has finally healed, but I'm sure that’s because I did what I was told* [P12]
Memory, attention and decision making	11	16	Attention	Attention not directed toward VLU management	*After I came back, I forgot how vital the compression was to the healing process. So, there was a period of time there where I wasn't treating the compression as seriously as I should have been. They need to have the compression on 24/7* [P19]

**TABLE 4:** Enablers to patients’ adherence to the evidence-based guideline recommendations for venous leg ulcer management.

Domains	No of participants mentioned	No of times referred	Main constructs	Main themes	Sample quotes
Knowledge	26	66	Knowledge	My past experience gave me knowledge	*A: No, I think, what's happens is it's almost like a little blister. That’s how it sort of starts, can, you can feel the flesh underneath it feel very sensitive and sort of bulging thingy.*
					*Q: And so when you felt that coming on, you went to the clinic or you went to your doctor?*
					*A: I usually go to the doctor* [P15]
				I conduct my own research	*Q: Was it easy to understand what they (clinicians) were telling you, or difficult to understand some of the things or concepts they talked about?*
					*A: Some of the things, I had to ask a second time as to what you mean, but mostly, it was understandable after reading up about ulcers and so forth and looking through the journals in the medical section of the library. Yeah, I went to the (University) Library and got some medical books out and had a bit of a read as to what bilateral ulcers are, treatment, and so forth.…I just wanted to check a couple of things I’d been told, but also look at some pictures of what happens, if I leave them for a lot longer than they were* [P25]
					*Q: Did you get any information given to you about what an ulcer is and what you need to do to help it improve to heal or any of that sort of information in the early days?*
					*A: We did discuss going to a vein specialist at the time, but nothing definite, but what I actually did, I came home and Googled information on it* [P28]
				Education received from healthcare professionals	*Q: When you started doing the compression or when you first were told to do compression, what information were you given by your doctors and nurses?*
					*A: Well I bought the Comprilan in and they showed me how to do it myself* [P19]
					*Q: Did you ask any questions the wound clinic staff, or did they give you anything printed, or were you able to recall what they told you?*
					*A: They told me moisturizing was really good, something that I probably had neglected too, rubbing moisturiser all over my feet, heels.*
					*Q: Why is that good for you?*
					*A: To stop any little cracks getting into your heels and getting an infection in there, making sure the shower is totally clean of bacteria, watching that you don’t walk around in bare feet, and keeping yourself really extra clean, fungal infections and all that sort of thing, we discussed* [P28]
					*Staff were very informative as to what I should be using to cut down on the infection, the bacterial load. They were more helpful in recommending where to buy things, obviously knowing that, as a public patient, it’s very hard to afford to buy top-line wound management kits. So, they had to refer me off to places where they aren’t very expensive.* [P29]
				Application of knowledge	*I was shown how to look after it (wound) and I think I'd find that, trying to think of the problem. Oh, I'm now qualified how to look after it. Like I know what I'm doing and I know what I'm looking at* [P05]
					*Q: And what do you believe helped your ulcer heal?*
					*A: I think the advice that I got from the clinic. I think also probably with my nursing background, understanding perhaps whereas maybe other nonmedical people perhaps don't fully understand how the body works* [P16]
					*I think if people haven’t had any background in science, for example, and can’t quite grasp what the medical profession are telling them, it would be useful for them to have someone explain it to them in general terms. That would probably apply to most people, they haven’t done science or chemistry and they can’t grasp what’s being done* [P24]
				I understood the rationale for using compression	*Yeah, I’d much rather have them (compression) not on when it’s 40 degrees. They get a bit warm in summer, but that’s a small price to pay for not spending another ten days in hospital* [P01]
					*Q: Would you consider keep using compression?*
					*A: Yeah, yeah, yeah. I mean, they’ve told me that I’ll be burdened with it for the rest of my life, and I’ve come to grips with that.*
					*Q: Do you know why? Even if it heals?*
					*A: I guess it’s blood flow, and that’s probably why I’m on the list for something to go back and see the vascular people again* [P02]
					*A: I went to see the compression sock I saw in (a specialist clinic). And he (the vascular specialist) told me that I must wear a compression sock on that leg.*
					*Q: Okay. And what did he why you had to wear it?*
					*A: Yes, because of poor circulation. And at that time I had the ulcer as well* [P05]
					*Q: Did you get information about why compression works?*
					*A: I guess so. I must have, but I do understand that it does help to keep the fluid out, and I do have a lot of problem with fluid getting down into that leg. And I know that the compression helps to relieve all that* [P07]
					*A: The bandages are awful especially when it's 40 degrees outside, it's the last thing you feel like wearing but you have to do it. Sorry, you just have to do it.*
					*Q: When you say you have to do it, why do you have to do it?*
					*A: Well, I had to do it because that was what I told would cure the ulcer... or get rid of the ulcer* [P14]
					*A: And that’s what they pushed at the wound clinic was compression, compression, compression.*
					*Q: And what did they tell you about it, why?*
					*A: To increase the blood flow to go back up, and to reduce the swelling. It’s not just what you put on the wound, it’s not just debriding. It’s because my feet were fat, swollen, so it was reducing the fluid in them, that’s why I wasn’t allowed to stand up for long periods of time, I had to keep my legs up to reduce the fluid and increase the venous flow* [P25]
Social influences	19	44	Social support	Family support	*My family helps out. Like I said, in terms of it just makes my life a little bit simpler to just get a lift to (the hospital)* [P13]
					*A: My son actually dresses it for me and he's almost turned into a nurse.*
					*Q: Yeah, you would with all of those experience.*
					*A: Alright. He does the dressings and then I've had two broken wrists, so I can't, I haven't got the strength in my hand to pull the elastic cotton stockings on and off. So, he puts them on and pulled them off for me. I'm very thankful that he is near me* [P15]
					*One of the reasons why we go with each other to appointments is to take on board all this information in case one misses something the other picks up on it. That's always been an important thing with us when we go to appointments. We'd both be there and sometimes some of the stuff can be a little overwhelming depending on what you're doing. If someone else asks the questions you might forget to ask you say, “Oh, I forgot to ask that question. It slipped my mind.” So, there is a benefit in both going to these things. …My wife basically did most of that because I couldn't put bandages on and all that sort of stuff. She was often worried she'd put them on a bit too firm. Basically, she did the best she could and she was confident on what she had done at home and the idea of doing it at home was to save a lot of trips* [P27]
					*A: One of my daughters who is a nurse practitioner in drug and alcohol. She had attended a lecture by Doctor (Name) because in her field they get a lot of patients. And she had told me how he was saying you don't use antibiotics, you don't use cream, you just use this, whatever the pad was and you know, Liofoam, and the three layers of compression stockings* [P16]
				Nurses’ support	*Q: I remember you saying before that you felt like they were a bit ugly the stockings. Was this a different type of stocking that looked better? Or you just decided to start wearing them? Or ...*
					*A: No, I wouldn’t go against the nurses, they know what they were doing. They’re experienced, and no, it gave me confidence, and what they said was right* [P04]
					*Q: Did you trust the nurses?*
					*A: Absolutely.*
					*Q: At both clinics?*
					*A: Yes. Ah, absolutely* [P30]
				Employers’ support	*It’s toward eight to ten years or so that I’ve been treated for the leg ulcers. And actually, and I just recall now, when I was working, I was officially working five days a week, but my department would let me actually work from home two days a week. So, I don’t - where I didn't have to go into the city, and that was because of the ulcer conditions being or beginning to develop at that time* [P09]
					*Q: You said that your boss was quite supportive. Were you able to talk to them about it? Did you feel what was going …*
					*A: Yes, I told them I couldn’t work, I was in hospital, “What’s your problem? Are you not well?” I said, “I’ve just got an intravenous drip because I’ve got an infection in my leg”, “You take as much time off as you need”, that sort of thing* [P28]
				Social Norms	*Q: Was this from casualty, after the antibiotics, or from the wound clinic?*
					*A: No, from casualty.*
					*Q: Before you got into the wound clinic appointment.*
					*A: But then when I went to the wound clinic, I saw people with things on their legs and bandages, and I thought, “Well, they must know what they’re doing here because there’s a lot of people with the same problem.”*
					*Q: Was that the first time you’d seen people with bandages on legs or thought it was …*
					*A: Well, yes, I’ve never noticed it before, but now I’m preoccupied and I see people in these tubigrips or whatever, swollen legs.*
					*Q: You know now.*
					*A: They’ve got one, he’s got one, I’ve got one too* [P28]
Beliefs about consequences	11	21	Consequence	Worried about health complications	*A: There’s a plastics specialist at the (Hospital) and a wound consulting team recommended a graft on both legs, and then elevation, rest, and hospital stay for seven to 10 days, then back dressings, and then home.*
					*Q: Okay. They’ve got a plan, and then everyone’s on to that. That sounds good.*
					*A: Correct.*
					*Q: How do you feel about that plan?*
					*A: It’s going to be daunting taking two months of bed rest, but at the end of the day, two months or no legs, take your pick* [P29]
					*Q: So, you've got the stocking now.*
					*A: And I wear them every day. They are an absolute pain in the butt to put on, they're a pain in the butt to take off. I can sort of get them on myself, but my husband does it for me on and off. So we bought an adapter and I've got, you know, an adapter that you put the stocking over. But I put them on every day and my legs don't swell anymore and you know, it's worth it. I didn't know how I'd go in the summer time, but I survive, which has been a pretty hot summer. And I hate the heat.*
					*Q: What made you keep persevering?*
					*A: Because my legs feel so comfortable and I figure, it's easier than going and having a varicose vein strip. Because I've had some surgery and I just don't want it anymore if I can avoid it. So my legs are very … and I'm not a small person. I'm very, very comfortable* [P16]
					*A: I also take a lot more care of my legs and my feet now than what I used to.*
					*Q: why is that?*
					*A: Because I don’t want to get any more ulcers* [P01]
					*I’m much more aware of preventive. If I get the cellulitis, like I had a bout of cellulitis two weeks ago, I s’pose, and in the past I might have been tending to been a bit slow in treating it. Now if I feel any warmth get into my legs, or my legs get at all sore, or start to swell a bit, I just go straight onto the antibiotics. Whereas, if in the past, I used to let it go a bit, the cellulitis would take hold, or my legs would tend to peel, all the skin would peel off them. And I’d finish up with a week or ten days in hospital on an antibiotic drip* [P01]
Emotions	2	3	Positive effect	It felt good when I was reassured	*A: I would like to know if it's going to get worse because, if I had known it's going to get worse. I could've been more prepared for just emotionally and mentally at least prepare for it getting worse rather than thinking that it's going to clear off and be okay. Whereas I think when it got to the point where it wasn't clearing up, and going to be okay. They should sort of say, well it's going to come down to a skin graft [P14]*
					*Q: Coming to see the podiatrist, how did that feel for you?*
					*A: It felt good. They were not judgmental. They unwrapped everything, and they said, “We can heal those, we can move forward. You don’t have to wear bandages the rest of your life.”*
					*Q: How did you feel when they told you that?*
					*A: Felt fantastic* [P29]
Environmental context and resources	5	10	Material resources	The wound clinic had superior resources	*Q: When the treatment you were having for your ulcer, while you were at the clinic, was it similar to the treatment you were having prior, like dressings and things like that?*
					*A: Well yeah, it was, but it was the type of dressing that they were using. It was the actual wound care product that they were putting on the skin graft. It was the product they were using before they put the Comprilan, the Comprilan pressure dressings are a bonus. The actual dressings they were putting on the wound rather than most of the time, in hospital, they put on some dressings, some gauze, and a bit of tape. Whereas the ladies at clinic, they’re using some designated dressing for that particular problem.*
					*Q: Yeah, that’s true, they have a big range too, don’t they?*
					*A: And they take one look at it, and in consultation with the Wound Care doctors there, and with your blood tests you’ve had done, they take a look and they say, “Well we need to use this.” And it’s this, out of about six or seven different products they could use. But it’s the right one for the job* [P01]
Beliefs about capabilities	10	15	Perceived competence	I can do compression	*Q: How confident would you say you feel about managing your stockings at home?*
					*A: Oh, very confident.*
					*Q: Is that from years of just having, you know, ulcers or of looking at different compression stockings or?*
					*A: Just I’ve been wearing them for a long time now, so that just doesn't bother me. You put them on* [P05]
					*Q: How competent do you feel managing your compression applications at home? Putting them on.*
					*A: Just got no problems. I've been doing it for so long now. It's just so dramatic, you know, because I now if I'd done them, I've tried not wearing them. Right. I'm a like feel dreadful [P06]*
					*Q: Well, I'll just jump back to compression. So did you do your own stockings and things at home and you felt confident to do all of your compression by yourself?*
					*A: Yes, I was fine. I would make sure when I went there then I showed them how I was doing it and also they'd see when they were taking marks, you know, they seemed pretty happy with what I was doing. I knew how to do it. It was, just the hassle of keeping it on, getting it off sometimes. Just all* [P14]
					*Q: So you felt comfortable enough sort of hinting at your doctor or asking or?*
					*A: well, it was probably the nurse because I was having visits to the nurse. I would call the doctor in if you know, because I was going twice a week. And doing it myself at home and the benefits of a nursing background. So I feel that I've put a lot of effort into looking after the two, once I was even that way to do it. So I was quite happy to change the dressings. If I changed them four times a day, I changed them. I just bought bulk supply* [P16]
Behavioral regulation	11	12	Action planning	Providing input that influences the care that is delivered at an organization (ie., GP clinic)	*A: It’s easier for them to do them because they come into the wound clinic and seen what’s going on, and then they’ve now managed it and they know what to look for when it starts to get smelly. They know what to look for when they start looking infected, and they know all the tell-tale signs. There might be an underlying infection.*
					*Q: How did that come about, that they got to get to the clinic and get educated?*
					*A: I invited them there one day because they needed to learn, as they’re going to be treating me more often than what* (the hospital) *was.”* [P29]
					*A: I mean, when they don't have the nurses on, like after work time or on the weekends or whatever, I'll sort of - you know, I have asked them: "can they?" and "how come?" and then like within a week, the owner of the clinic in (Location) said, “No, we need nurses here on - at night time all the time,” so. Because the nurses are rostering themselves. Yeah, with picking up kids and all that sort of stuff, and then he said, “Yeah well, it's getting on.” Like I didn't complain. I mean, to the reception people I said: “Ah, this is getting a bit hard. I work here, so it's too hard to get to during lunch break, or whatever,” and they said, “Ah, it's just how it is.” And then within a week, it had changed* [P31]
					*Q: Did you feel you had any other options when they were discussing the stockings with you or things you could…*
					*A: I don’t think they gave me any options. They just said, well this is what you have to, probably they said this is what we'll do. It's like my feet the podiatrist surgeon said to me, you need to get specially made boots so that your foot doesn't, trying to hold your foot from collapsing anymore. So, I went, got them. The cost me $1,900 each oh, but I did it. The next fortnight when I went back to him, he nearly fell off his chair because someone had actually done what he'd suggested would be best for their feet.*
					*Q: So you did decide … the costs didn’t put you off?*
					*A: Well, no, we've always put health first and so if you know for both my husband and I and for our children. That we had private health, you know, if you need to go to the doctor … we’ve done it, you know what I mean? We've spent the money and okay, it's a lot of money sometimes, but it's not quite unnecessarily miserable because you don't want to spend any money on getting something done, for your own benefit* [P16]
			Self-monitoring	Persistence and patience	*A: But once they put those compression things on, I think the pain went to about 12 out of 10. But that was after I went home. Do you know what I mean? But I could feel and I could see the benefits because my legs during the day would swell, my ankles would swell. And then night time they would go down. But those stockings were pushing the fluid, they were sort of almost washing the leg from inside every time. I was changing those dressings three and four times a day. Sometimes it was so much, you know, each day or whatever. And then after a week or 10 days, the pain in them started to ease off. I persevered* [P16]
					*Q: What made you keep coming back if in those initial stages you were finding the pain?*
					*A: I just made myself come back!* [P25]
Memory, attention and decision making	3	4	Memory	I have a good memory	*Q: Do you feel quite confident and capable at the appointments of asking questions or going prepared with questions or taking in all the information at the time?*
					*A: Yes, that’s not a problem.*
					*Q: Do you take notes at all?*
					*A: No, I’ve got a very good memory.* [P28]

### Knowledge

Twenty-six participants (83%) discussed enablers that pertained to knowledge. Key themes included understanding the rationale for treatment, sources of information and ways these enablers were beneficial to treatment adherence. These included three main categories: 1) knowledge from personal experience: “Since my wounds have healed I’ve basically done everything myself. Now I know what goes on … I’ve learnt little bit and pieces” [P11]; 2) information provided by a healthcare professional: “Staff were very informative as to what I should be using to cut down on the infection, the bacterial load.” [P29]; and 3) information from personal search: “I went to a University Library and got some medical books out and had a bit of a read as to what bilateral ulcers are, treatment, and so forth” [P25]. Eighteen participants said that they used Internet to get information on VLUs, while thirteen acknowledged that they relied only on information provided by clinicians as the most trustable source of information. A few participants had limited information technology skills, and perceived this as a barrier to getting information *via* the Internet.

Twenty-one participants (67%) reported they understood the reason for compression: “I do understand that it does help to keep the fluid out, and I do have a lot of problem with fluid getting down into that leg. And I know that the compression helps to relieve all that” [P07]. For example, one of the participants said that this understanding allowed them to accept compression as a necessary treatment: “I’ll be burdened with it for the rest of my life, and I’ve come to grips with that” [P02].

Knowledge and acceptance optimized participants’ adherence with compression. P13 outlined his acceptance to wear compression for a past health condition and understood the benefit.

I’ve had to use compression stockings in the past for different reasons, including just general swelling in my feet and ankles. So, when they mentioned compression I obviously knew what they were talking about … It’s just when you understand how the venous ulcers work then it like obvious … To me it was like this is probably the best way to heal it. So, they didn’t really have to sell it to me [P13].

Understanding how compression aided healing was a key enabler for participants when deciding whether to adhere to clinician’s treatment suggestions:

Q: And what made you decide to use the compression? A: Well it was the desire of the medicos to increase the vascular flow. The valves are leaking in the veins, that unfortunately prevents all your blood returning to your heart. So, they put the compression on to force the blood back up, as much as possible [P24].

Conversely, participants reported that complex, inconsistent or incorrect information provided by clinicians all served as barriers to VLU treatment. Compression, for example, was a particular area where participants reported confusion. Participants highlighted that sometimes this confusion originated from the clinician who was treating their wound: “I don’t really think the GP (General Practitioner) knew that much about ulcers” [P14]. Participants said they did not conduct research of their own and instead relied on qualified health professionals: “If they said jump, I jumped” [P16]. As such, when P17 was asked why they stopped wearing compression, they reported that it was because they had been told by a vascular surgeon that it was no longer necessary.

When asked about whether there was information or follow up provided about long term prophylactic compression to prevent future ulceration, P17 reported that she’d assumed she didn’t need compression because she was now able to exercise: “Well, the next thing was to go on my walking program, so I just thought that’s the price to pay for not having compression stockings? Because she (clinician) said, to develop the muscles, to strengthen the muscles in my legs, to help your circulation. I just assumed that’s why I didn’t need compression stockings.”

When participants felt that they understood treatment, they were able to troubleshoot barriers. For example, P20 said that the initial stockings suggested by her GP caused her a lot of discomfort. When she went to the wound clinic, however, they provided her with education and explored methods that would prevent the discomfort, leading to compression adherence:

When I first got the compression stocking and the first ulcer healed, I stopped wearing it because it used to rub a bit on my leg. But since the second one, I haven't stopped wearing it. Because when I went back and they said: “Well, you should be wearing it” I said “Well it rubs!” Apparently, I wasn’t told, I was supposed to have something done to it so it wouldn’t rub... just putting it on the leg. So, the second lot of nurses were more informative than the first [P20].

One participant explained that though they understood that clinics were busy, they really appreciated when they were given VLU education: “I know they’re really busy as they are; and I understand that. I think it probably would have helped, if they had explained it a little bit more as to what was actually happening … ” [P14].

### Social Influences

Participants discussed social influences as an enabler to adhering to treatment plans under the TDF constructs social support and social norms. Participants reported improved adherence when they saw other people in the wound clinic undergoing similar treatment: “when I went to the wound clinic, I saw people with things on their legs and bandages, and I thought, “Well, they must know what they’re doing here because there’s a lot of people with the same problem” [P28]. This quote could also be interpreted as an expression of trust in health professionals.

Social support was a very important enabler to accessing VLU care. Family members were highlighted as the key members providing social support. The following caregiving activities increased patients’ adherence with the VLU treatment recommendations: assistance with dressing or/and compression application; assistance with computer technology to access health information; explanation of medical information and terminology; taking to medical appointments; helping to make treatment decisions; and encouraging treatment. Social support was also provided by formal carers and (community) nurses who visited patients at home. Participants also relied on supportive work-related environment with employees providing flexible arrangements for the patients to attended frequent medical appointments.

Some participants raised body image concerns related to wearing compression: “Out of vanity, I don’t like wearing (compression)—I don't like having one thing on my legs. I look like a— yeah, it’s self—a self-conscious thing, I suppose you call it” [P25]. One participant acknowledged health professionals’ support, including related to the body image issues: “I said to him, “How am I going to work at a shopping centre with a big, fat thing like this on my leg?” He said, “I was dealing with a lady that sells fur coats in the top end of (Street name), and she could manage it, so you can too” [P28].

### Beliefs About Consequences

Understanding the benefits of treatment and consequences for non-adherence were the key factors driving participants’ adherence. In particular, understanding the consequences of not using compression was an important factor influencing participants’ adherence. Participants reported a belief that failure to act quickly would cause their condition to worsen. Those who experienced VLU for the first time explained that they sought out medical advice because they could see that their wound was getting worse, and they feared that an inaction or a delay would lead to serious health consequences: “I have to look for a solution because it’s not getting any better, it’s getting worse”, and you become quite desperate” [P28].

Participants who had experience with VLUs before, knew that VLUs were difficult to heal, necessitating prompt action:

A: I had it (a VLU) when I was 30, so I knew what it was, and—yeah, I wanted to get on top of it as quickly as I could, so. Q: The one that you had when you were 30, what was that one like? A: Yeah, that lasted—ah, for three years. It was terrible. Because I went to GPs over and over and over, and getting dressings and all that sort of stuff [P31].

Another participant commented that they were very meticulous with following self-care instructions provided by their healthcare practitioner; driven by fear that they will develop multiple ulcers: “I’m better now, because I don’t want to get them [VLUs] again, I worry more about what I do. I don’t touch my legs, if they’re feeling puffy, I put them up” [P25].

### Emotions

Participants described a range of emotions that influenced VLU treatment adherence. These ranged from feeling daunted or overwhelmed by their diagnosis; to feeling desperate and distressed as their VLU worsened: “I got desperate” [24]. One participant explained that she was very concerned by her wound: “I started to get really concerned, and I thought, ‘I have to look for a solution because it’s not getting any better, it’s getting worse’, and you become quite desperate because your legs have to hold you, and I was terrified someone was going to hit me or bump me.” [P28]. Pain also made participants feel distressed: “I talked about my pain … I couldn’t function before. I was probably yelling at (Name) a lot and was just grumpy, full stop. But once I got that on, I could actually do stuff, I could go places and enjoy life a bit more. It is pretty depressing being in so much pain [P25].

### Environmental Context and Resources

Patients reported a number of environmental and financial issues that interfered with VLU management. Transport related issues, such as living a distance from their local practice and/or limited parking spaces were discussed by eight participants: “It is quite a problem getting from here to (the clinic) by public transport” [P12]. Proximity and ability to meet appointment times greatly impacted on the patient’s ability to attend their scheduled appointments. For example, P31 found that he was unable to continue working due to the barriers imposed by managing his wound as his appointments were so frequent, time consuming and disruptive. Other barriers under this domain included hot weather and related discomfort when wearing compression on hot days: “Well, the bad points are that it’s constrictive, and it’s hot in the summer when you’re trying to get to sleep is hard.” [P02].

Lastly financial issues related to VLU management were discussed by all patients. They were related to absence from work due to medical appointments, the cost of transport and parking, and the out of pocket cost of compression hosiery and adjunct therapies: “There’s no comparison of the Comprilan in terms of getting compression. They sell it for like 70 bucks a pair as well. They’re so expensive” [P19].

### Beliefs About Capabilities

Participants reported that agency to perform wound care, especially compression, was an enabler to self-care. Some participants were unable to adhere to evidence-based interventions due to limited mobility and physical constraints:

Q: Would you say that your immobility prevents you from doing any sort of exercise or anything that the GP might … (have recommended)?

A: It inhibits that, yes. I’ve got to use crutches, and sometimes I get a bit worn under the arms and so forth. Over the last six months, they’ve recommended that I elevate my leg during the day. So, it’s complicated, because I can only get on and off the bed when there’s a carer present with me. I can’t get either on or off myself, and so I have some limited funds to have carers, and I have them seven days a week, but for two or three hours at a time [P09].

Nine participants reported they could apply compression and wound dressings on their own at home. P19 (Table 4) explained that he was shown how to perform compression and change wound dressings at home. Being capable to perform these procedures on his own, he was not required to attend the clinic as frequently. Other participants also reported beliefs about one’s own capabilities was an enabler for adherence to management plans.

### Behavioral Regulation

Participants discussed behaviors that related to their behavioral regulation, under the constructs Generating Alternatives and Self-monitoring. For example, one participant who was in full-time employment had made a request at their primary care to have nurses available afterhours, who would change dressings and apply compression. This example of generating alternatives, meant that participants found it easier to adhere to VLU management and meet their care plan.

Five participants discussed the importance of patience and persistence. For example, when P16 was asked why she persevered with her frequent dressings and compression, she described that though her wound was “a nuisance” and “a frustration,” she was “determined to stick it out.” Furthermore, participants could see the link between persisting with treatment, and subsequent wound healing:

Q: What do you think made your ulcer heal? Or what do you think helped it the most?

A: Well, I think that silver stuff you put on it, I think that's a great thing. I think persistence, and actually doing as I was told with it, to be perfectly honest [P30].

Though care tasks were identified as being arduous, participants believed their wound would heal faster when they demonstrated resilience.

### Memory, Attention, and Decision-Making

Older participants reported barriers that pertained to memory, attention and decision making. They found it difficult to recall aspects of their care and VLU management. They forgot who has made their VLU diagnosis, if diagnostic imaging was performed in the initial consultation and what information was provided: “I can’t remember getting any paperwork, but that doesn’t mean anything. I could have reams of it in a place somewhere at home.”—[P30]. The latter presents a barrier for patients to perform self-management of their VLU. When they are unable to recall the specifics of their treatment plan (or the rationale for doing so) then they may be less likely to comply with their care directives.

Some people found it difficult to remember to keep their legs elevated. For example, after explaining key details of needing to elevate her leg, P30 explained that they frequently forgot to do this:

A: (General practitioner) explains “You’re supposed to have your legs up all the time,” but I can’t—I wouldn’t say no … The answer would be yes, but I don’t do it.

Q: Why is that?

A: I don ’t know. I just don’t think of it, I suppose [P30].

One participant described that they often had many self-care tasks prescribed to them that were often monotonous, and thus they found their attention was not directed to their self-care:

I guess, sometimes, when you’re getting ready for bed, if you’re tired and you’ve got about four or five things to do, they seem monotonous. Then I’ll get to bed and, if you are really tired, that seems like a load on your mind. But other times, you might say get them over and done with; and it’s all over in about five minutes [P27].

This participant indicated that a sense of persistence was important for overcoming the feeling of burdensome monotony associated with self-care.

## Discussion

We analyzed patients’ experiences, with the aim of identifying the barriers and enablers influencing patients’ adherence to venous leg ulcer treatment recommendations in primary care settings. There is a paucity in the literature examining strategies to enhance health-promoting behaviors in patients with venous disease (Miller et al., 2014). Through utilizing the TDF, however, researchers can explain factors that influence patient adherence or non-adherence to VLU treatment. In this article, we specifically examined patient adherence to the main components of their venous leg ulcer management plan, including compression use, leg elevation, nutrition and exercise. In addition, patients also discussed their experience in scheduling and attending appointments—a key aspect of achieving optimal outcomes for their VLU. Compression adherence was of particular importance, given that current literature reflects that adherence to compression use on daily basis is as low as 28% (Atkin, 2019). We found that the most frequently discussed domains related to VLU patients’ adherence to VLU management plans included Knowledge, Social Influences, Beliefs about Consequences, Emotions, Environmental Context and Resources, Beliefs about Capabilities, Behavioral Regulation and Memory Attention and Decision Making (Tables 3, 4).

**FIGURE 4:**
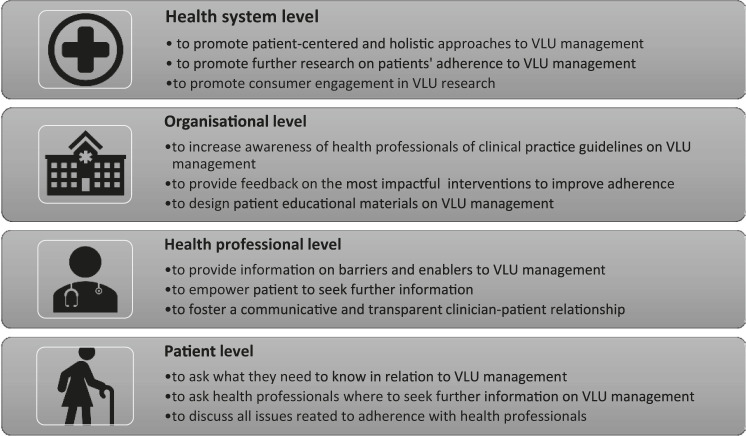
Strategies to improve adherence to the venous leg ulcer treatment recommendations.

Participants often did not understand the significance of continued use of compression; and discontinued compression once healed. This is unsurprizing, as the particular age demographic in our study (older adults) often prefer to acquire health information from a GP or other clinicians (Turner et al., 2018) rather than engage in self-directed inquiry through the use of the Internet (Chaudhuri et al., 2013). A few participants received either incorrect or inconsistent information from their clinicians (a nurse, a doctor and a vascular surgeon) in this regard. In some instances, participants did not get any health information on VLU management at all. This most certainly appears to be a missed opportunity for clinicians to build patients’ agency and provide an opportunity for shared decision making (Weller et al., 2021). Previous studies have examined the relationship between the delivery of health information, the development of greater patient health literacy and improved adherence to health-promoting behaviors (Miller et al., 2014). This is consistent with our findings, as participants explained that once they understood the reason for their treatment, they were more motivated to adhere to treatment (Please refer to Figure 4 for this and other suggestions on how to improve VLU treatment adherence). The proposed topics for health education may include the discussion of the main barriers to wearing compression therapy, body image issues, family support, and consequences of not wearing compression. It is pivotal that a GP, who first diagnoses a patient with a VLU, and the nurse, who changes dressings and applies compression provide a thorough assessment during consultation to empower patients to seek further information.

Other barriers to adherence to the VLU management plans include uncontrolled pain or discomfort; body image issues pertaining to societal norms, inadequate social support and competing commitments. These multiple factors need to be considered in order to change patients’ behavior and improve their adherence to VLU treatment. Fostering a communicative and transparent clinician-patient relationship has been found to be mutually beneficial (Janamian et al., 2016). Clinicians may benefit from feedback on adherence interventions that are most impactful for the patients. Patients benefit from the opportunity having their opinions and experiences considered, and to be able to troubleshoot issues that impede VLU treatment adherence, particularly their adherence to compression (Castro et al., 2016). A patient centred approach may serve to improve the evidence-practice gap in VLU management that exists across primary care (Weller et al., 2020; Weller et al., 2020). A recent study determined that patient centred care in the management of chronic wounds led to greater patient satisfaction, increased patient knowledge, enhanced patients’ adherence to VLU management, and improved quality of life (Gethin et al., 2020). Further research into the role of clinicians in VLU patient centred care may be warranted; as would identifying suitable strategies to foster engagement of patients in research and policy development (Weller et al., 2020).

## Conclusion

Our article demonstrates the value in exploring the patient experience of adhering to evidence-based treatment of VLU. Though patient non-adherence has been described as challenging for clinicians, the issue is multifaceted and complex. There is a need to further explore factors that influence VLU patients’ behaviors and provide an opportunity to plan interventions that best support both patients and clinicians in a shared decision-making capacity. The findings from this article will be relevant to clinicians involved in management of patients with venous leg ulcers, as their support is crucial to patients’ treatment adherence. Consultation with patients about VLU treatment adherence is an opportunity for clinical practice to be targeted and collaborative. This process may inform guideline development.

### Adequacy of the Selected Theory

The applied TDF (Atkins et al., 2017) was a useful approach to providing a theory-driven analysis to identify barriers and enablers to patients’ adherence to VLU treatment. The identified target behaviors of VLU patients may help to inform future implementation strategies in primary care settings (Phillips et al., 2015). However, these findings should be interpreted with caution. Qualitative approach, including theory-driven analysis aiming to identify barriers and enablers is frequently used in behavior change and implementation research. It is important note that researchers applying this approach may easily overlook the attribution of behavior (Sabini et al., 2001) or failure to behave as expected or prescribed, which may influence trustworthiness (Elo et al., 2014). Consideration should always be given to the attitude-behavior or intention-behavior gap (Godin et al., 2005).

In this study, some of the participants have mentioned that they have complete trust to their clinicians and health information they provide, nevertheless they did not follow their recommendations once compression was prescribed. Further, the participants discussed various factors that they believed had influenced their adherence to treatment. For example, they discussed that they did not use compression because of the body image-related issues, discomfort during hot weather, limited understanding the benefits of wearing compression and consequences of not wearing compression, the lack of family support with wearing compression stockings, and other barriers that fit well with the TDF constructs and domains. However, most of the participants did not wear compression because it was not prescribed to them by their GP, which was a real barrier. After prolonged suboptimal treatment in primary care, which has had no effect on VLU healing, they were referred to the specialist tertiary clinic, where compression was prescribed.

It is important to note that the TDF was developed for understanding the behavior of healthcare professionals related to implementation of evidence-based guidelines in clinical practice (Michie et al., 2005). We utilized this theory to explain patients’ adherence to VLU treatment, aiming to bring patients’ perspective on VLU management and to complement our study on the use of the VLU CPG recommendations in primary health care settings located in Melbourne metropolitan and rural Victoria, Australia (Weller et al., 2020).

As we already mentioned, our interview guide consisted of open-ended questions loosely matching the TDF domains (2017 version), and did not cover all 14 TDF domains. Therefore, the collected data are largely influenced by what questions the participants were asked, limiting the “richness” of data that could have been obtained using the qualitative approach alone (Phillips et al., 2015).

The theory-driven deductive analysis itself may limit the interpretative process, leaving no space for ideas that are not related to the theoretical domains of the TDF to be elicited (McGowan et al., 2020). By using the TDF domains as first-level codes and the TDF constructs as secondary level codes there is a danger that data are forced into the codes, limiting the interpretation.

Although we quantified how often or by how many participants references are made to specific constructs and domains, it is important to note that these numbers are highly influenced by the way the interviews were conducted, e.g., we much more explicitly asked for experiences and thoughts regarding compression adherence rather than the other VLU treatment recommendations; and, this was not identical in all interviews. This, of course, is in accordance with qualitative methodology, but one of the reasons why counting how often or by how many participants references are made to has limited value.

### Study Limitations and Recommendations for Research

As patients with VLUs require complex treatment, comprising a number of different evidence-based approaches, it can be expected that barriers and enablers playing an important role in the several measures differ, as what they appeal to or need, indeed, differs. In this study, we focused predominantly on patients’ adherence to compression therapy as it is the mainstay treatment identified by the VLU CPG. Future research about VLU patients’ adherence to exercise, leg elevation and nutrition recommendations would be beneficial. Further studies may also explore individual factors that influence VLU patients’ behaviors related to adherence to compression therapy. For example, VLU patients’ body image issues related to VLUs and wearing compression garments may be an area to be explored in greater depth. Studies that explore patients’ understanding the consequences of not wearing compression and not following other recommendations may also be of value to patients and clinicians to optimize healing outcomes in primary care.

We explored barriers and enablers that influence patients’ adherence to VLU treatment using a qualitative inquiry. Future studies on patient adherence to VLU treatment may identify predictors and extent of patient adherence to the VLU treatment recommendations, using validated questionnaires about patients’ adherence to health recommendations. Findings of these studies may provide information on the effectiveness of patient care management and outcomes, and identify patients with higher risk for poor adherence to inform and evaluate targeted interventions.

## Data Availability

The original contributions presented in the study are included in the article/Supplementary Material, further inquiries can be directed to the corresponding author.
